# New Anti-inflammatory Cyclopeptides From a Sponge-Derived Fungus *Aspergillus violaceofuscus*

**DOI:** 10.3389/fchem.2018.00226

**Published:** 2018-06-14

**Authors:** Jingtang Liu, Binbin Gu, Lianjuan Yang, Fan Yang, Houwen Lin

**Affiliations:** ^1^Research Center for Marine Drugs, State Key Laboratory of Oncogenes and Related Genes, Department of Pharmacy, Renji Hospital, School of Medicine, Shanghai Jiao Tong University, Shanghai, China; ^2^The Fungal Reference Laboratory of Shanghai Dermatology Hospital, Shanghai, China

**Keywords:** sponge-derived fungus, *Aspergillus violaceofuscus*, cyclic peptides, structural characterization, anti-inflammatory

## Abstract

Three new cyclic peptides including a cyclic tetrapeptide (**1**), an aspochracin-type cyclic tripeptide sclerotiotide L (**2**) and a diketopiperazine dimer (**3**), have been isolated from the ethyl acetate extract of a marine sponge-derived fungus *Aspergillus violaceofuscus*. The structures of all compounds were unambiguously elucidated on the basis of HRESIMS, 1D and 2D NMR spectroscopic data, MS/MS experiments and chemical methods. Compounds **1** and **3** showed anti-inflammatory activity against IL-10 expression of the LPS-induced THP-1 cells with inhibitory rates of 84.3 and 78.1% respectively at concentration of 10 μM.

## Introduction

As a class of important metabolites from marine-derived organisms, cyclic peptides are extensively present in marine tunicate (Ireland et al., 1982), sponge (Zhang et al., 2010), algae (Xu et al., 2008), bacteria (Teta et al., 2017), fungi (Bao et al., 2013), etc., and often these cyclopeptides possess rare molecular skeleton (Fukuhara et al., 2015). Moreover, due to versatile biological functions including antineoplastic (Ireland et al., 1982), antimicrobial (Teta et al., 2017), anti-inflammatory (Randazzo et al., 2001), antitubercular (Daletos et al., 2015) and histone deacetylase inhibitory activities (Gu et al., 2007), the cyclic peptides have received enduring attention of organic chemists, biologists and pharmacologists. The structures of cyclic peptides may contain unusual amino acids or be modified by methylation (Jang et al., 2017), acetylation, lipidation (Luo et al., 2014), and sulfuration (Fukuhara et al., 2015). These characteristics are playing a vital role in the interactions with relevant bioactive targets (Sieber and Marahiel, 2005; Raaijmakers et al., 2010).

**Figure 1:**
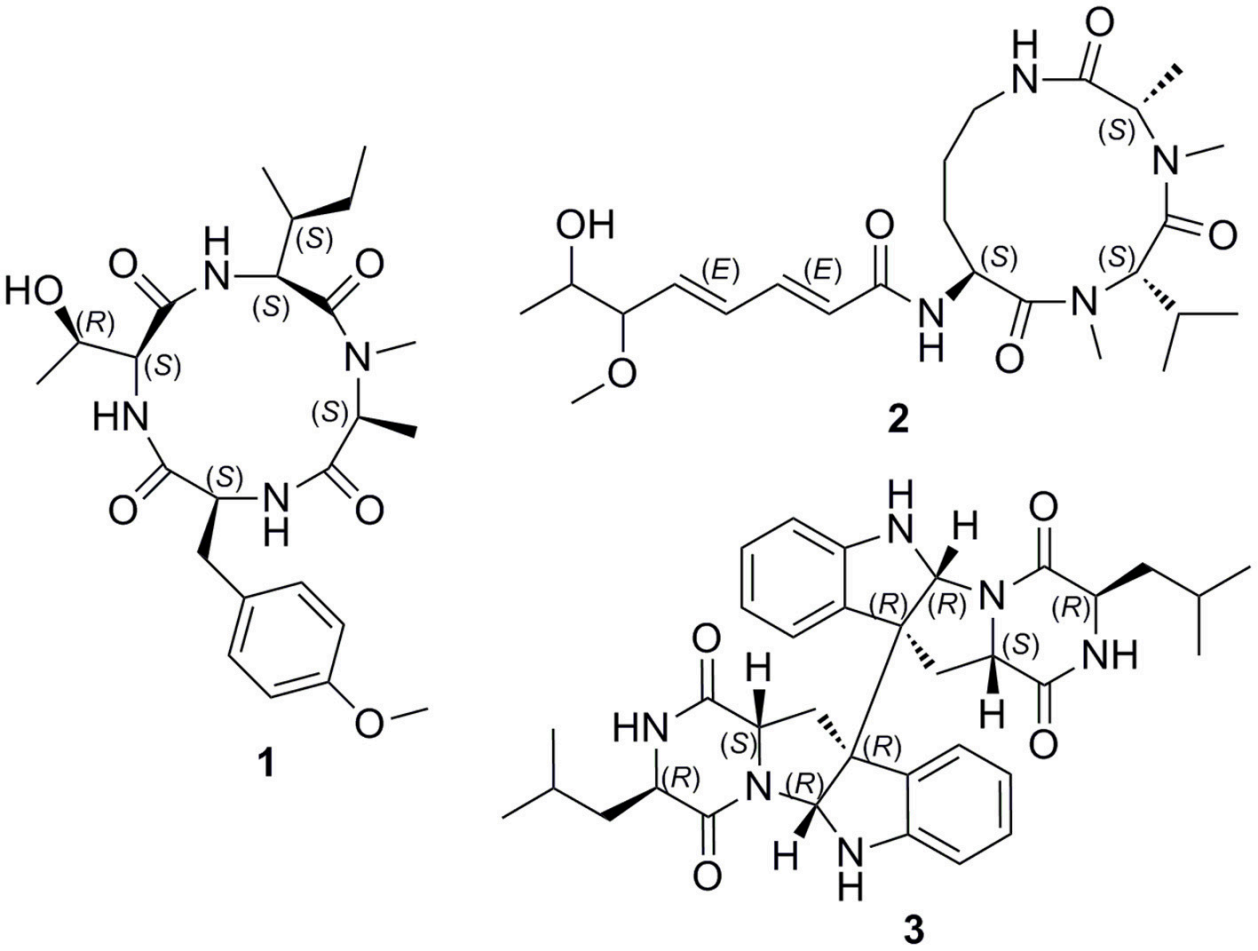
Structures of compounds **1-3**.

The sponge-derived fungi have been proven to be a prolific source of cyclic peptides (Amagata et al., 2006; Yu et al., 2008). In previous search for structurally unique cyclic peptides from marine sponge-derived fungus *Nigrospora oryzae* PF18 collected off the Xisha Islands in the South China Sea, we have identified a series of new cyclohexadepsipeptides oryzamides A–C (Ding et al., 2016). As part of our continuing quest for new bioactive molecules, chemical investigation of secondary metabolites of the fungus *Aspergillus violaceofuscus* from the marine sponge *Reniochalina* sp. resulted in the identification of three new cyclopeptides, including a cyclic tetrapeptide violaceotide A (**1**), an aspochracin-type cyclic tripeptide sclerotiotide L (**2**), and a new diketopiperazine dimer (**3**) (Figure 1). Herein, the isolation, structure elucidation and anti-inflammatory studies of the three new cyclic peptides were described.

## Materials and methods

### General experimental procedures

Optical rotations were determined on a Rudolph research analytical autopol VI polarimeter with a 1 dm length cell at room temperature. UV spectra were performed on a Persee TU-1950 UV-VIS spectrophotometer. The NMR spectra were recorded on a Bruker AMX-600 instrument. HRESIMS data were obtained on a Waters Xevo G2-XS Q-Tof mass spectrometer. Reversed-phase HPLC was performed on Waters X-Bridge C18 (5 μm) columns with a Waters 1525 separation module equipped with a Waters 2998 photodiode array detector. MPLC was accomplished using a Interchim PuriFlash 450 chromatography system. Silica gel 60 (200–300 mesh; Yantai, China), Sephadex LH-20 (18–110 μm, Pharmacia Co.) and ODS (50 μm, YMC Co.) were used for column chromatography.

### Fungal strain and fermentation

The fungus *Aspergillus violaceofuscus* was isolated from the inner part of the marine sponge *Reniochalina* sp. collected from the Xisha Islands in the South China Sea. The sample was deposited at the Research Center for Marine Drugs, School of Medicine, Shanghai Jiao Tong University. This strain was identified based on the morphology analyze and ITS gene sequencing (GenBank accession No. FJ491681).

The strain was cultivated on potato dextrose agar at 28°C for 7 days. Large scale fermentation was carried out in 50 erlenmeyer flasks (2 L) each containing 80 g of rice and 120 mL of distilled H_2_O with 0.3% (m/v) peptone. Each flask was inoculated with 20 mL of cultured broth and incubated under static conditions at room temperature for 40 days.

### Extraction and isolation

The fermented substrate was exhaustively extracted with ethyl acetate to provide the residue (26.0 g) after removal of the organic solvent under reduced pressure.

The EtOAc extract was fractionated by vacuum liquid chromatography on silica gel (200–300 mesh) using CH_2_Cl_2_/MeOH gradient elution (500:1–0:1, v/v) to give eleven fractions A–K. Fraction H (6.1 g) was separated by column chromatography (CC) over Sephadex LH-20 eluted with MeOH to afford subfractions H1–H9. Subfraction H3 (1.5 g) was applied to medium pressure liquid chromatography (MPLC) on ODS, eluted with a gradient of 10 to 100% (v/v) MeCN in H_2_O, to give H3A–H3F. H3D was subjected to reversed-phase HPLC with an elution of 55% MeOH in H_2_O to give **1** (2.0 mL/min, *t*_R_ = 23.0 min, 8.6 mg). H3C (31.3 mg) was purified by semi-preparative reversed-phase HPLC eluted with 18% MeCN in H_2_O to yield **2** (2.0 mL/min, *t*_R_ = 37.7 min, 2.0 mg). Subfraction H2 (1.82 g) was applied to MPLC on ODS, eluted with a gradient of 10 to 100% (v/v) MeCN in H_2_O, to give Fr. H2A–H2H. Fraction H2F (169 mg) was then purified by semi-preparative RP-HPLC eluted with 37% MeCN in H_2_O resulted in the isolation of **3** (2.0 mL/min, *t*_R_ = 30.5 min, 1.8 mg).

Violaceomide A (**1**): white amorphous powder; [α]25 D −230 (*c* 0.6, MeOH); UV (MeOH) λ_max_ (log ε) 220 (3.26), 276 (1.31) nm; HRESIMS m/z 477.2719 [M + H]^+^ (calcd for C_24_H_37_N_4_O_6_, 477.2713); ^1^H and ^13^C NMR data, Table 1.

**Table 1:** ^1^H (600 MHz) and ^13^C NMR (150 MHz) Data for **1** in Pyridine-*d*_5_.

**Position**	**δ_C_**	**δ_H_, mult. (*J* in Hz)**	**Position**	**δ_C_**	**δ_H_, mult. (*J* in Hz)**
N-Me-Ala			12	65.3, CH	4.37, dd (8.9, 2.4)
1	173.6, C		13	68.1, CH	4.78, m
2	55.1, CH	4.74, m	14	22.2, CH_3_	1.40, d (6.3)
3	17.0, CH_3_	1.46, d (7.1)	12-NH		7.20, brs
4	30.8, CH_3_	3.32, s	O-Me-Tyr		
Ile			15	173.8, C	
5	171.4, C		16	55.9, CH	4.47, m, overlapped
6	55.7, CH	5.14, dd (10.0, 7.6)	17	35.7, CH_2_	3.81, m; 3.63, m
7	37.6, CH	2.37, m	18	132.7, C	
8	17.6, CH_3_	1.16, d (6.5)	19/23	131.9, CH	7.31, d (8.0)
9	25.2, CH_2_	2.00, m; 1.42, m	20/22	114.9, CH	7.03, d (8.2)
10	12.4, CH_3_	0.94, t (7.4)	21	159.6, C	
6-NH		8.80, brs	24	55.9, CH_3_	3.78, s
Thr			16-NH		9.30, brs
11	173.2, C				

Sclerotiotide L (**2**): pale yellow amorphous powder; [α]25 D −92 (*c* 0.5, MeOH); UV (MeOH) λ_max_ (log ε) 216 (3.84), 258 (3.98) nm; HRESIMS m/z 481.3042 [M + H]^+^ (calcd for C_24_H_41_N_4_O_6_, 481.3026); ^1^H and ^13^C NMR data, Table 2.

**Table 2:** ^1^H (600 MHz) and ^13^C NMR (150 MHz) Data for **2** in CDCl_3_.

**Position**	**δ_C_**	**δ_H_, mult. (*J* in Hz)**	**Position**	**δ_C_**	**δ_H_, mult. (*J* in Hz)**
Ala			10-NH		6.53, d (7.2)
1	171.5, C		11	28.6, CH_2_	2.39, m; 1.60, m
2	55.2, CH	4.59, q (7.1)	12	21.9, CH_2_	1.66, m; 1.57, m
3	17.0, CH_3_	1.51, d (7.1)	13	39.7, CH_2_	3.38, m; 3.06, m
*N*-CH_3_	29.9, CH_3_	3.06, s	13-NH		5.68, brs
Val			Fatty acid		
4	169.2, C		1′	164.8, C	
5	58.8, CH	5.11, d (10.5)	2′	124.2, CH	5.92, d (15.0)
6	27.0, CH	2.43, m	3′	140.3, CH	7.23, dd (15.0, 10.8)
7	20.0, CH_3_	0.92, d (6.3)	4′	132.1, CH	6.36, dd (15.4, 11.0)
8	18.0, CH_3_	0.74, d (6.8)	5′	137.7, CH	6.00, dd (15.4, 7.8)
*N*-CH_3_	30.4, CH_3_	2.95, s	6′	85.5, CH	3.62, dd (7.8, 3.7)
Orn			7′	69.5, CH	3.90, dd (6.5, 3.7)
9	173.1, C		8′	18.0, CH_3_	1.12, d (6.5)
10	49.7, CH	4.98, t (7.2)	9′	57.1, CH_3_	3.32, s

Compound (**3**): White powder. [α]25 D +530.0 (*c* 0.3, MeOH); UV (MeOH) λ_max_ (log ε) 239 (3.58), 300 (1.55) nm; HRESIMS m/z 597.3177 [M + H]^+^ (calc. for C_34_H_41_N_6_O_4_ 597.3189). ^1^H and ^13^C NMR data, Table 3.

**Table 3:** ^1^H (600 MHz) and ^13^C NMR (150 MHz) Data for **3** in CDCl_3_.

**Position**	**δ_C_**	**δ_H_, mult. (*J* in Hz)**	**Position**	**δ_C_**	**δ_H_, mult. (*J* in Hz)**
2/2′	80.5, CH	4.94, s	12/12′	37.2, CH_2_	3.18, dd (14.0, 9.1)
3/3′	59.7, C		13/13′	168.5, C	
4/4′	130.0, C		14/14′		5.93, s
5/5′	124.9, CH	7.34, d (7.5)	15/15′	56.3, CH	3.77, m
6/6′	119.9, CH	6.81, t (7.5)	16/16′	168.2, C	
7/7′	129.8, CH	7.15, t (7.6)	17/17′	41.8, CH_2_	1.48, m; 2.82, dd (14.0, 8.6)
8/8′	110.3, CH	6.65, d (7.9)	18/18′	24.5, CH	1.64, m
9/9′	148.8, C		19/19′	23.1, CH_3_	0.89, d (6.5)
11/11′	55.8, CH	3.99, t (8.8)	20/20′	21.3, CH_3_	0.87, d (6.5)

### Advanced Marfey's analysis of compound 1

Compound **1** (1 mg) were hydrolyzed in HCl (6 M; 1 mL) for 18 h at 110°C. The solutions were then evaporated to dryness and redissolved in H_2_O (200 μL). The aqueous hydrolysate was added with 1% (w/v) solution of 1-fluoro-2,4-dinitrophenyl-5-L-leucinamide (L-FDLA, 100 μL) in acetone and 1 M NaHCO_3_ (40 μL). After treating at 45°C for 90 min, the reactions were quenched by the addition of HCl (1 M, 40 μL). Appropriate amino acid standards were treated with L-FDLA and D-FDLA as described above and yielded the L-FDLA and D-FDLA standards. Marfey's derivatives of **1** was subjected to UPLC-MS selected ion chromatography on a reversed-phase column (Waters ACQUITY HS T3 column; 1.8 μm, 2.1 × 100 mm) with a linear gradient from 10 to 60% aqueous CH_3_CN containing 0.1% formic acids over 18 min and their retention times were compared with those from the authentic standard derivatives.

### Advanced Marfey's analysis of compound 2

Compound **2** (1 mg) were hydrolyzed in HCl (6 M; 1 mL) for 20 h at 110°C. The solutions were then evaporated to dryness and redissolved in H_2_O (200 μL). The aqueous hydrolysate was divided into two equal portions. One portion was treated with 1% (w/v) solution of 1-fluoro-2,4-dinitrophenyl-5-D-leucinamide (D-FDLA, 100 μL) in acetone and 1 M NaHCO_3_ (40 μL). The second portion was treated with a racemic mixture of a 1% (w/v) solution of 1-fluoro-2,4-dinitrophenyl-5-D-leucinamide (D-FDLA, 50 μL) in acetone, 1% (w/v) solution of 1-fluoro-2,4-dinitrophenyl-5-L-leucinamide (L-FDLA, 50 μL) in acetone, and 1 M NaHCO_3_ (40 μL). Both mixtures were heated at 45°C for 90 min and the reactions were quenched by the addition of HCl (1 M, 40 μL). The aliquots were subjected to HPLC-MS selected ion chromatography on a reversed-phase column (Waters XBridge C18 column; 5 μm, 4.6 × 250 mm; 1.0 mL/min) with a linear gradient from 10 to 80% aqueous CH_3_CN containing 0.1% formic acids over 30 min according to the advanced Marfey's method. The retention times and ESIMS product ions (*t*_R_ in min, *m*/*z* [M + H]^+^) of the D-FDLA mono-derivatized amino acids in the hydrolysate of the first portion were observed to be Orn (13.1, 427.5), *N*-Me-Ala (20.4, 398.5), and *N*-Me-Val (24.3, 426.4), while the reaction with racemic D/L-FDLA in the second portion gave rise to two peaks for each corresponding amino acid moiety. The retention times and ESIMS product ions (*t*_R1_/*t*_R2_, min, *m/z* [M + H]^+^) were observed to be Orn (13.1/14.1, 427.5), *N*-Me-Ala (20.1/20.4, 398.5), and *N*-Me-Val (22.1/24.3, 426.4). Consequently, the absolute configuration of the amino acids in the hydrolysate of **2** was confirmed as L-Orn, *N*-Me-L-Ala, and *N*-Me-L-Val.

### Marfey's analysis of compound 3

Compound **3** (0.4 mg) was dissolved in 6 N HCl (1 mL) and heated at 110°C for 24 h. Then, the solvent was evaporated under reduced pressure and resuspended in 50 μL of H_2_O. The hydrolysates were treated with 200 μL of 1% (w/v) 1-fluoro-2,4-dinitrophenyl-5-L-leucinamide (FDLA) in acetone and 40 μL of 1.0 N NaHCO_3_. The reaction mixtures were heated at 45°C for 2 h, cooled to room temperature, and then neutralized with 40 μL of 1 N HCl. Standard D-Leu and D/L-Leu were derivatised in a similar fashion separately. The derivatives of the hydrolysates and the standard amino acids were analyzed by LC-MS selected ion chromatography on a reversed-phase column (Waters XBridge C18 column; 5 μm, 4.6 × 250 mm; 1.0 mL/min) with a linear gradient from 10 to 100% aqueous CH_3_CN containing 0.1% formic acids over 30 min. The retention times for FDLA derivatives of standard D-Leu and L-Leu were 21.8 and 18.7 min, respectively, while this for FDLA derivatives of compound **3** were 21.8 min.

### Anti-inflammatory assay

THP-1 (a human acute monocytic leukemia cell line) cells (CCTCC) were maintained in RPMI-1640 supplemented with 10% (v/v) FBS and 0.05 mmol/L 2-mercaptoethanol at 37°C in a 5% CO_2_ and humidified environment. THP-1 cells (5 × 10^5^/mL) were differentiated using 160 nmol/L PMA for 36 h. Differentiation of PMA-treated cells was enhanced by removing the PMA-containing media and the cells were incubated in FBS free, fresh RPMI 1640 for a further 12 h, and then stimulated with compounds or/and LPS at the indicated concentrations and time periods.

Cytokines IL-6, IL-10, MCP-1, and TNF-α in the culture media of THP-1 cells treated with 10 μM compounds or/and 0.1 μg/mL LPS were determined by flow cytometry using the Human Inflammation Cytometric Bead Array (CBA) according to the instruction of the manufacturer (BD Biosciences, San Jose, CA, USA). Cytokine levels were measured on a FACSCalibur flow cytometer (BD Biosciences Pharmingen). The concentrations were assessed by using FCAP Array software.

## Results and discussion

**Figure 2:**
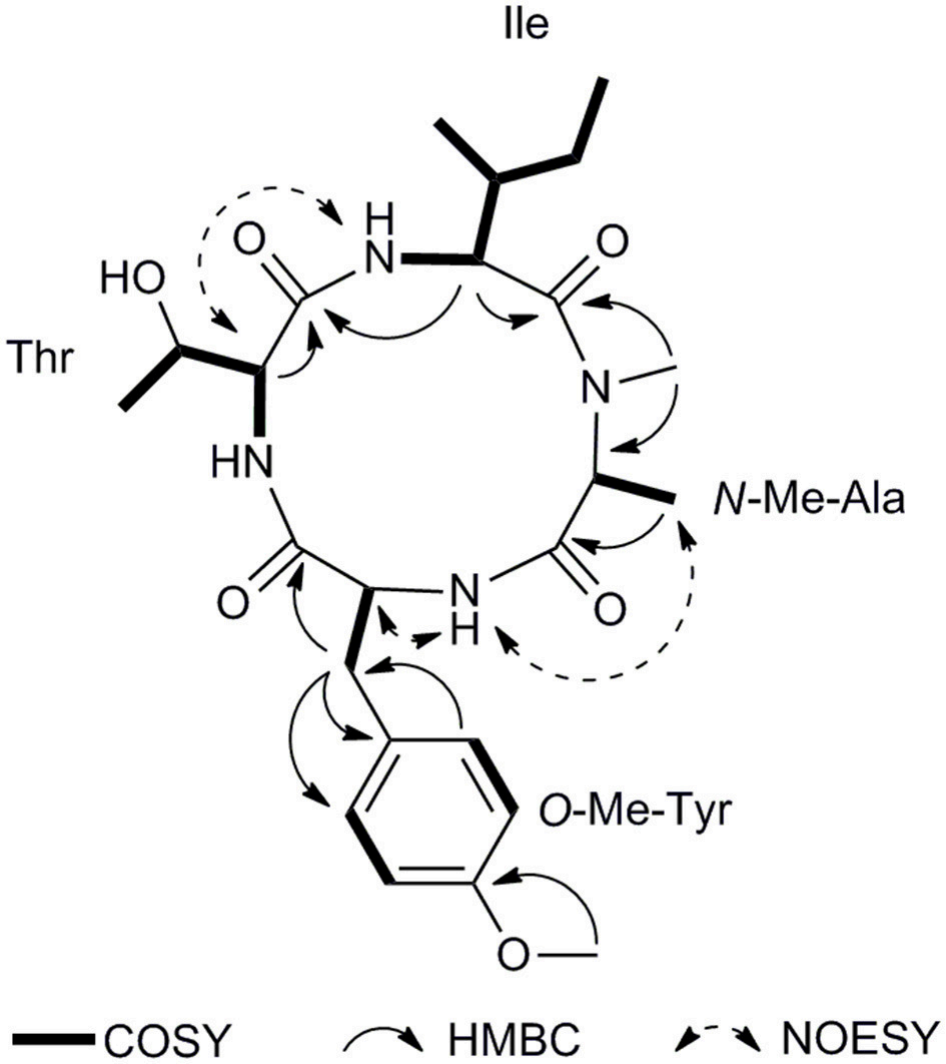
Key COSY, HMBC, and NOESY correlations of **1**.

**Figure 3:**
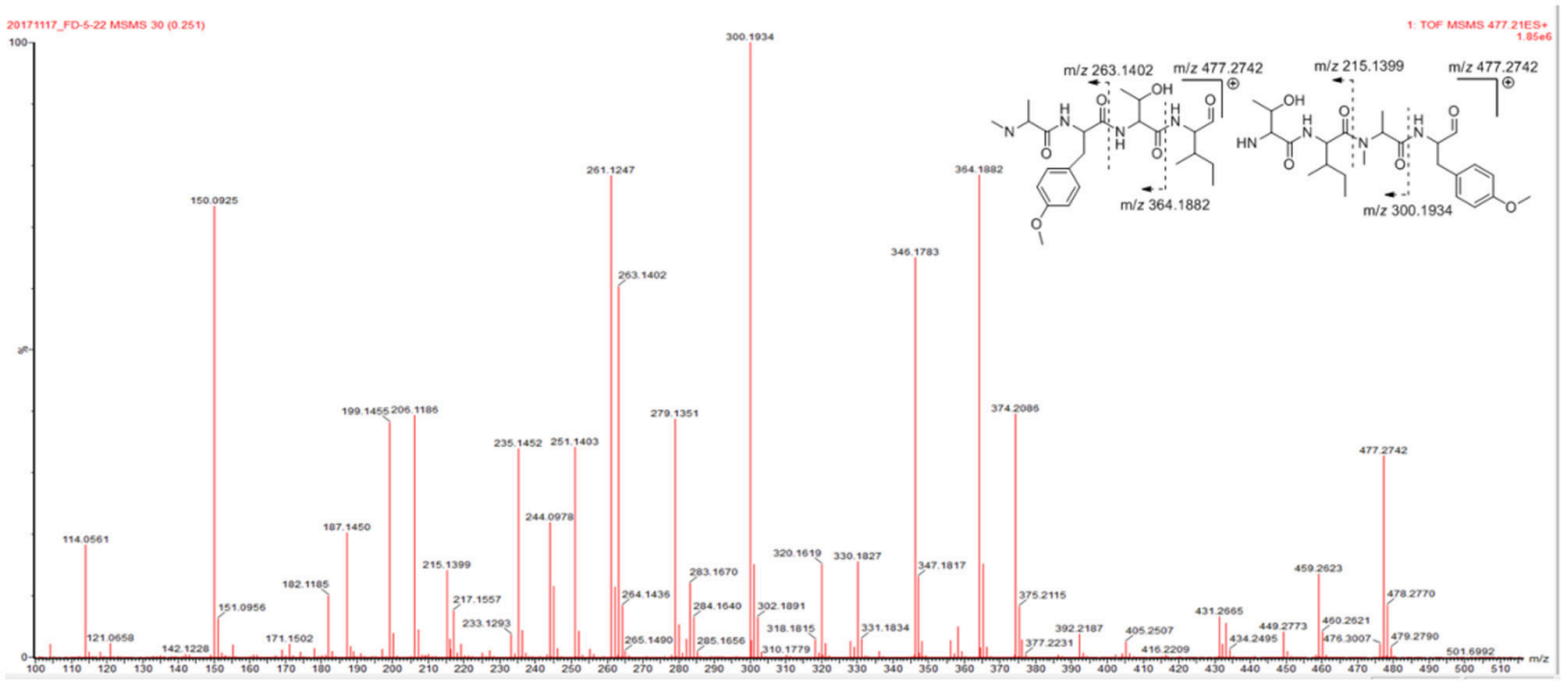
MS/MS spectrum of **1**.

Compound **1** was isolated as a White, amorphous powder, which possessed a molecular formula of C_24_H_36_N_4_O_6_ deduced from the pseudomolecular ion peak at *m/z* 477.2719 [M + H]^+^ in its HRESIMS (Supplementary Figure 19). The signal distribution pattern observed in the ^1^H and ^13^C NMR spectrum (pyridine-*d*_5_) (Supplementary Figures 1–2), which showed three exchangeable amide NH signals (δ_H_ 9.30, 8.80, 7.20), four amide carbonyls (δ_C_ 173.8, 173.6, 173.2, and 171.4), four characteristic α-methine signals (δ_H/C_ 5.14/55.7, 4.74/55.1, 4.47/55.9, and 4.37/65.3) and one *N*-methyl (δ_H/C_ 3.32/30.8), indicated the characteristic of a peptide. Combined analysis of the 2D NMR spectra (Supplementary Figures 3–6) revealed the structures of four amino acid residues, including alanine (Ala), isoleucine (Ile), threonine (Thr), and one tyrosine (Tyr). An HMBC correlation from 4-*N*-CH_3_ (δ_H_ 3.32) to Ala C-2 (δ_C_ 55.1) indicated that the Ala residue was *N*-methylated. The 24-OCH_3_ linked to the benzene ring at C-21 was supported by the HMBC correlation from H_3_-24 (δ_H_ 3.78) to C-21 (δ_C_ 159.6). The assignment of the amino acid sequence was carried out by a combination of HMBC, NOESY (Figure 2), and MS/MS analysis. The HMBC correlations from *N*MeAla H_3_-4 (δ_H_ 3.32) to Ile C-5 (δ_C_ 171.4) and from Ile H-6 (δ_H_ 5.14) to Thr C-11 (δ_C_ 173.2), suggesting a partial sequence of *N*MeAla-Ile-Thr. The NOESY correlation between *O*MeTyr NH (δ_H_ 9.30) and *N*MeAla H_3_-3 (δ_H_ 1.46) extended this sequence to *O*MeTyr-*N*MeAla-Ile-Thr. The 9° of unsaturation and the molecular formula suggested that **1** was a cyclic peptide. Therefore, the cyclic tetrapeptide ring was closed between *O*MeTyr and Thr. In addition, the amino acid sequence of **1** was confirmed by mass fragmentation analysis using a quadrupole-time-of-flight (Q-TOF) tandem mass spectrometer (Figure 3). Consequently, the planar structure of **1** was elucidated as a cyclic tetrapeptide with the sequence cyclo-(Thr-*O*-MeTyr-*N*-MeAla-Ile).

The absolute configurations of the amino acids were determined by the advanced Marfey's method after acid hydrolysis (Fujii et al., 1997). Compound **1** was hydrolyzed and then derivatized with L-FDLA. The UPLC-MS comparison between Marfey's derivatives of the hydrolysate of **1** and appropriate amino acid standards assigned the L configurations for Thr, Tyr, *N*-Me-Ala, and Ile (Supplementary Figure 23). The final structure of **1** was elucidated as cyclo-[L-Thr–L-*O*-Me-Tyr–L-*N*-Me-Ala–L-Ile] and named as violaceotide A.

**Figure 4:**
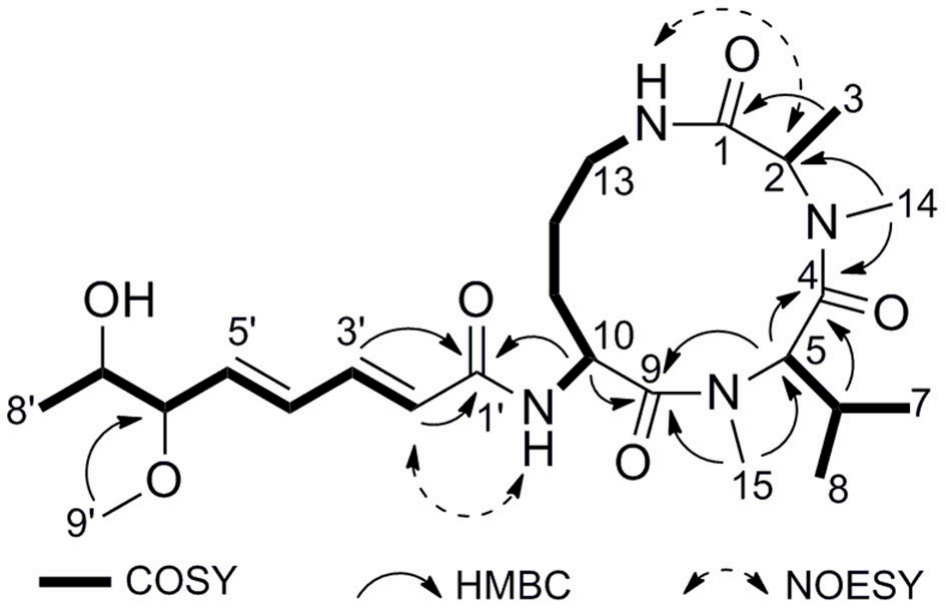
Key COSY, HMBC, and NOESY correlations of **2**.

Compound **2** was isolated as a pale yellow amorphous powder. The molecular formula can be determined as C_24_H_40_N_4_O_6_ by HRESIMS ion peak at m/z 481.3042 [M + H]^+^ (calcd for C_24_H_41_N_4_O_6_, 481.3026) (Supplementary Figure 20). The ^1^H NMR spectrum of **2** showed an amide NH proton (δ_H_ 6.53), two *N*-methyl protons (δ_H_ 2.95 and 3.06), and three characteristic α-methine signals (δ_H_ 4.59, 4.98, and 5.11), indicating a tripeptide structure. The ^13^C NMR spectrum exhibited a total of 24 carbon resonances, including four amide carbonyl carbons, ten methine carbons, three methene carbons, and seven methyl carbons. The obvious difference in the NMR spectra between compound **2** and sclerotiotide H (Zheng et al., 2010) was the appearance of an additional *O*-methyl group at δ_H_ 3.32 (H-9′) and δ_C_ 57.1 (C-9′). The HMBC correlations (Figure 4) from H_3_-9′ to C-6′ (δ_C_ 85.5) revealed that the *O*-methyl was linked to fatty acid chain at position 6′ (Supplementary Figures 7–12). The geometries of the Δ^2^′, 3′ and Δ^4^′, 5′ olefins were identified as 2′*E* and 4′*E*, by the proton spin coupling constants of $J_{\text{H}-2^{\text{′}},\text{H}-3^{\text{′}}}$ (15.0 Hz) and $J_{\text{H}-4^{\text{′}},\text{H}-5^{\text{′}}}$ (15.4 Hz). In order to determine the absolute configurations of the amino acid residues of **2**, advanced Marfey's method was utilized (Fujii et al., 1997). HPLC-MS analysis of derivatives of the hydrolysates with D-FDLA and D/L-FDLA indicated that the amino acids were *N*Me-L-Ala, *N*Me-L-Val, and L-Orn (Supplementary Figure 24). Thus, compound **2** was elucidated as (2′*E*,4′*E*)-*cyclo*-[(*N*Me-L-Ala)-(*N*Me-L-Val)-(*N*_α_-6′-methoxy-7′-hydroxyocta-2′,4′-dienoyl-L-Orn)] and named as sclerotiotide L.

Compound **3** was isolated as a white amorphous powder. Its molecular formula was determined as C_34_H_40_N_6_O_4_ on the basis of the pseudomolecular ion peak at m/z 597.3177 [M + H]^+^ (calcd for C_34_H_41_N_6_O_4_, 597.3189) in the HRESIMS (Supplementary Figure 21), requiring 18 degrees of unsaturation. The NMR spectra of **3** revealed 17 carbon signals, indicating that **3** would be a symmetric homodimer which was further confirmed by its half MS fragment ion peak at m/z 298.2 (Supplementary Figure 22).

**Figure 5:**
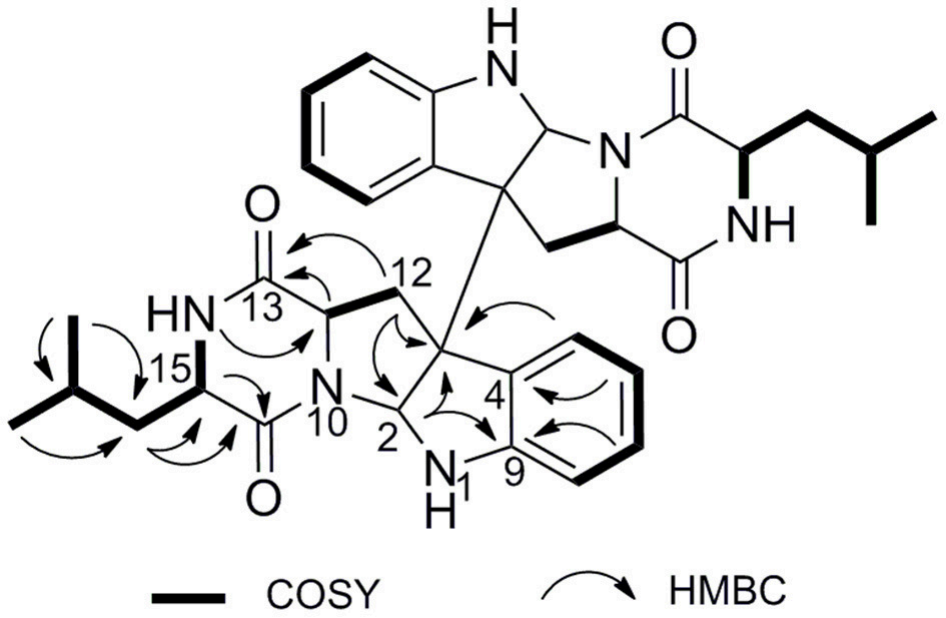
Key HMBC and COSY correlations of **3**.

In the ^1^H NMR spectrum, four aromatic signals at δ_H_ 7.34 (d, *J* = 7.5 Hz, H-5/H-5′), 7.15 (t, *J* = 7.6 Hz, H-7/H-7′), 6.81 (t, *J* = 7.5 Hz, H-6/H-6′), and 6.65 (d, *J* = 7.9 Hz, H-8/H-8′) and a proton at 4.94 (s, ^1^H, H-2/H-2′) were observed, which suggested a indoline moiety.^16^ In addition, two methyl signals were also observed at δ_H_ 0.89 (d, *J* = 6.5 Hz, H_3_-19/H_3_-19′) and 0.87 (d, *J* = 6.5 Hz, H_3_-20/H_3_-20′). The ^13^C NMR spectrum exhibited a total of 17 carbon resonances, including five quaternary carbons, eight methine carbons, two methene carbons, and two methyl carbons. Two ^13^C NMR resonances at δ 168.5 (C-13/C-13′) and δ 168.2 (C-16/C-16′) were characteristic signals of lactam carbonyls. The HMBC correlations from H-17 (δ_H_ 1.48) to C-15 (δ_C_ 56.3), and C-16 (δ_C_ 168.2) and COSY correlations of H-14 (δ_H_ 5.93)/H-15 (δ_H_ 3.77)/H-17/H-18 (δ_H_ 1.64)/H-19 and H-18/H-20 established a leucine unit (Figure 5). A tryptophan moiety can be concluded from the ^1^H NMR signals and the key HMBC correlations from H-12 (δ_H_ 3.18) to C-2 (δ_C_ 80.5), C-3 (δ_C_ 59.7), C-11 (δ_C_ 55.8), and C-13, from H-2 to C-3, and C-9 (δ_C_ 148.8). Ultimately, extensive analysis of the 2D NMR data and comparison of the spectroscopic data with the reported literature (Ovenden et al., 2004) allowed identifying the planar structure of **3** as shown (Supplementary Figures 13–18).

**Figure 6:**
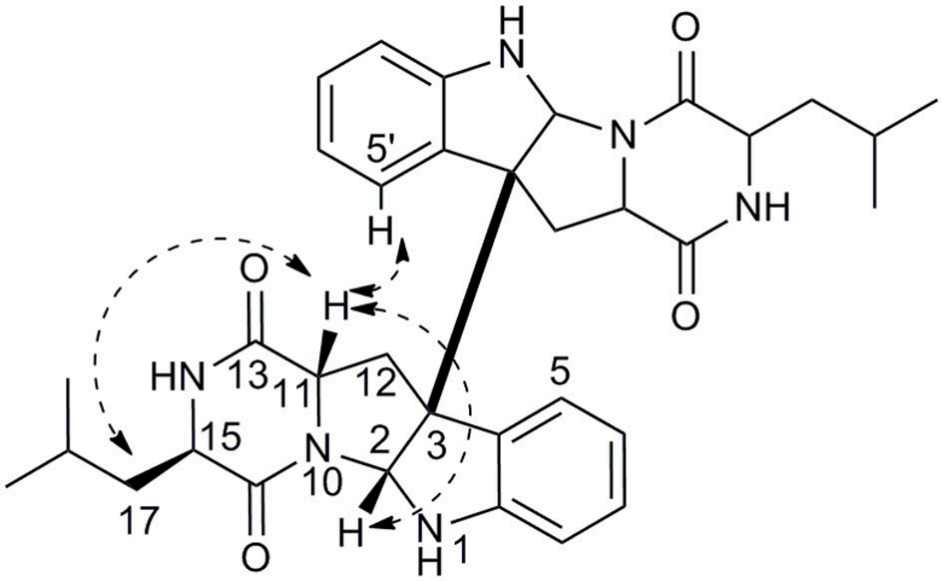
Key NOESY correlations of **3**.

The relative configurations of **3** were deduced from the observed NOESY correlations (Figure 6). The key NOESY correlations of H-11/H-17 and H-11/ H-2, indicated that these protons were on the same face. The NOESY correlations of H-5′ with H-11 and H-11 with H-2 suggested *cis*-fused ring junction at C-2 and C-3. Marfey's method (Cho et al., 2018) was employed to determine the absolute configuration at C-15. Compound **3** was hydrolyzed and derivatized with L-FDLA and analyzed by LC-MS to the establishment of absolute configuration of Leu residues. By comparing the retention times of authentic standards of L- and D- forms of Leu, the hydrolysate was identified to contain a unit of D-Leu (Supplementary Figure 25). Therefore, the absolute configurations of **3** were assigned as 2*R*, 3*R*, 11*S*, 15*R* (Figure 1).

To the best of our knowledge, compound **3** has the same planar structure as a unnamed and ambiguous compound without any spectroscopic data or optical rotation published on a patent (Masashi et al., 1995). Our compound possesses the different stereochemistry with it. Therefore, **3** was reported as a new diketopiperazine dimer herein.

Compounds **1–3** were evaluated their inhibitory activities against the production of four cytokines levels in the serum of human acute monocytic leukemia cell line THP-1 by using the human inflammation cytometric bead array (CBA) assay (Table 4). The cytokines, including IL-6, IL-10, MCP-1, TNF-α in this study, are pivotal mediators that contribute to inflammation and various related diseases (Liu et al., 2017; Wu et al., 2017). Notably, treatment of THP-1 cells by LPS showed a significant elevation in the secretion of the cytokines (*P* < 0.01). Results showed that the THP-1 cells pretreated with compounds **1** and **3** showed a significant decrease in the LPS-induced expression of IL-10 with inhibitory rates of 84.3 and 78.1% (*P* < 0.01), respectively. These compounds did not show cytotoxicity against THP-1 cells after 24 h treatment.

**Table 4:** The inhibitory rates of compounds **1-3** against the cytokines expression of LPS-induced THP-1 cells at concentration of 10 μM.

**Compound**	**IL-6 (%)**	**IL-10 (%)**	**MCP-1 (%)**	**TNF-α (%)**
1	45.9	84.3	32.9	64.2
2	28.0	23.6	40.5	61.5
3	51.2	78.1	40.0	63.1

## Conclusions

From the marine sponge-derived fungus *Aspergillus violaceofuscus*, three new cyclic peptides were obtained. Aspochracin-type cyclic tripeptide sclerotiotide L (**2**) and a diketopiperazine dimer (**3**) showed anti-inflammatory activity against IL-10 expression of the LPS-induced THP-1 cells, which indicated that the marine sponge-derived microorganism are a fertile source of compounds with novel structures and significant bioactivities.

## Author contributions

JL, BG, and LY: performed the experiments; JL: identified the structures and analyzed the data; HL and FY: conceived and designed the experiments; JL and FY: wrote the paper. All authors listed have approved the work for publication.

### Conflict of interest statement

The authors declare that the research was conducted in the absence of any commercial or financial relationships that could be construed as a potential conflict of interest.
