# Supplementary material for: New Anti-inflammatory Cyclopeptides From a Sponge-Derived Fungus Aspergillus violaceofuscus
Source: Front Chem. 2018 Jun 14;6:226. doi: 10.3389/fchem.2018.00226 (PMC6010530; doi:10.3389/fchem.2018.00226)
Supplement: Supplementary file 1 [file Data_Sheet_1.DOCX]

Supplementary Material

New Anti-inflammatory Cyclopeptides from a Sponge-derived Fungus *Aspergillus violaceofuscus*

Jingtang Liu^†^, Binbin Gu^†^, Lianjuan Yang, Fan Yang*, Houwen Lin*

*** Correspondence:** Fan Yang: yang-fan@sjtu.edu.cn, Houwen Lin: franklin67@126.com

**Supplementary Figure 1.** ^1^H NMR spectrum (600 MHz) of compound **1** in Pyridine-*d*_5_.

**Supplementary Figure 2.** ^13^C NMR spectrum (150 MHz) of compound **1** in Pyridine-*d*_5_.

**Supplementary Figure 3.** ^1^H-^1^H COSY spectrum of compound **1** in Pyridine-*d*_5_.

**Supplementary Figure 4.** HSQC spectrum of compound **1** in Pyridine-*d*_5_.

**Supplementary Figure 5.** HMBC spectrum of compound **1** in Pyridine-*d*_5_.

**Supplementary Figure 6.** NOESY spectrum of compound **1** in Pyridine-*d*_5_.

**Supplementary Figure 7.** ^1^H NMR spectrum (600 MHz) of compound **2** in CDCl_3_.

**Supplementary Figure 8.** ^13^C NMR spectrum (150 MHz) of compound **2** in CDCl_3_.

**Supplementary Figure 9.** ^1^H-^1^H COSY spectrum of compound **2** in CDCl_3_.

**Supplementary Figure 10.** HSQC spectrum of compound **2** in CDCl_3_.

**Supplementary Figure 11.** HMBC spectrum of compound **2** in CDCl_3_.

**Supplementary Figure 12.** NOESY spectrum of compound **2** in CDCl_3_.

**Supplementary Figure 13.** ^1^H NMR spectrum (600 MHz) of compound **3** in CDCl_3_.

**Supplementary Figure 14.** ^13^C NMR spectrum (150 MHz) of compound **3** in CDCl_3_.

**Supplementary Figure 15.** ^1^H-^1^H COSY spectrum of compound **3** in CDCl_3_.

**Supplementary Figure 16.** HSQC spectrum of compound **3** in CDCl_3_.

**Supplementary Figure 17.** HMBC spectrum of compound **3** in CDCl_3_.

**Supplementary Figure 18.** NOESY spectrum of compound **3** in CDCl_3_.

**Supplementary Figure 19.** HRESIMS spectrum of compound **1**.

**Supplementary Figure 20.** HRESIMS spectrum of compound **2**.

**Supplementary Figure 21.** HRESIMS spectrum of compound **3**.

**Supplementary Figure 22.** ESI-MS spectrum of compound **3**.

**Supplementary Figure 23.** Advanced Marfey’s analysis of **1**.

**Supplementary Figure 24.** Advanced Marfey’s analysis of **2**.

**Supplementary Figure 25.** Marfey’s analysis of **3**.


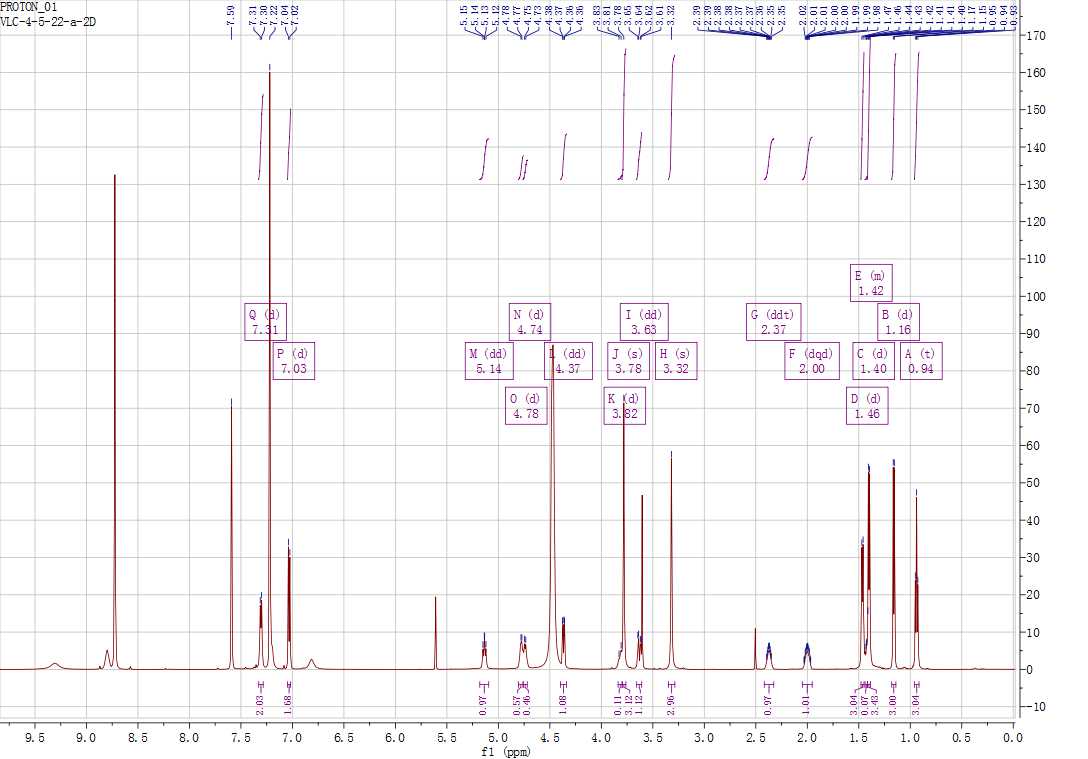


**Supplementary Figure 1.**


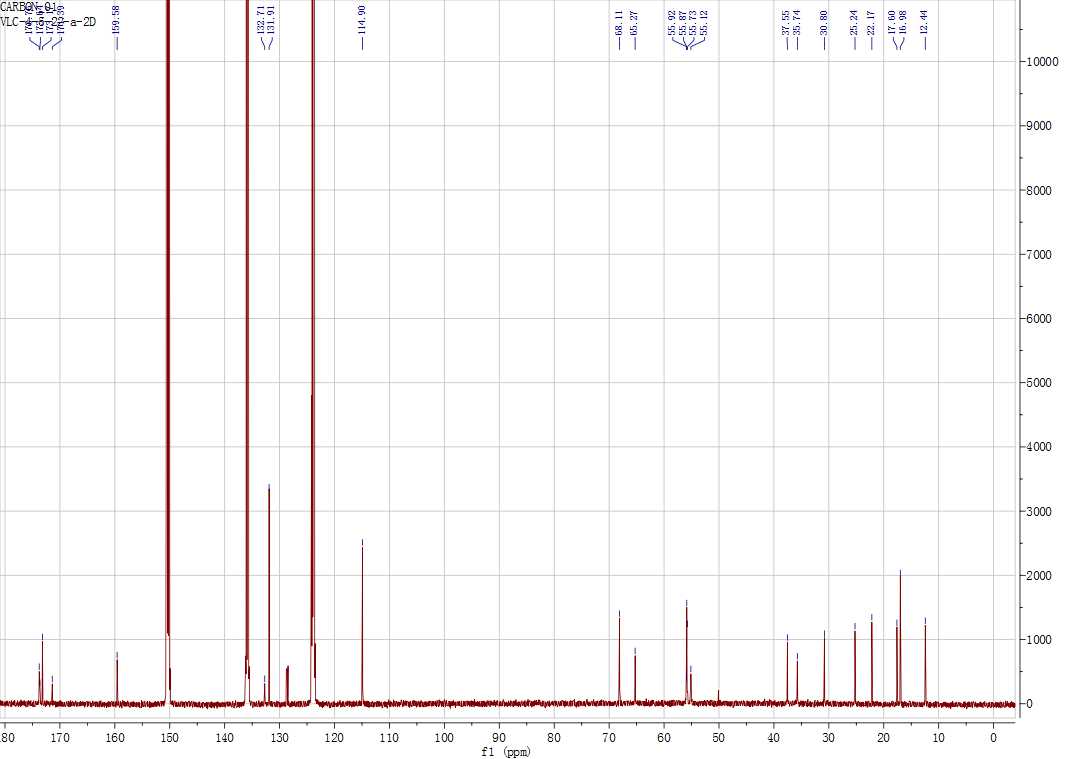


**Supplementary Figure 2.**


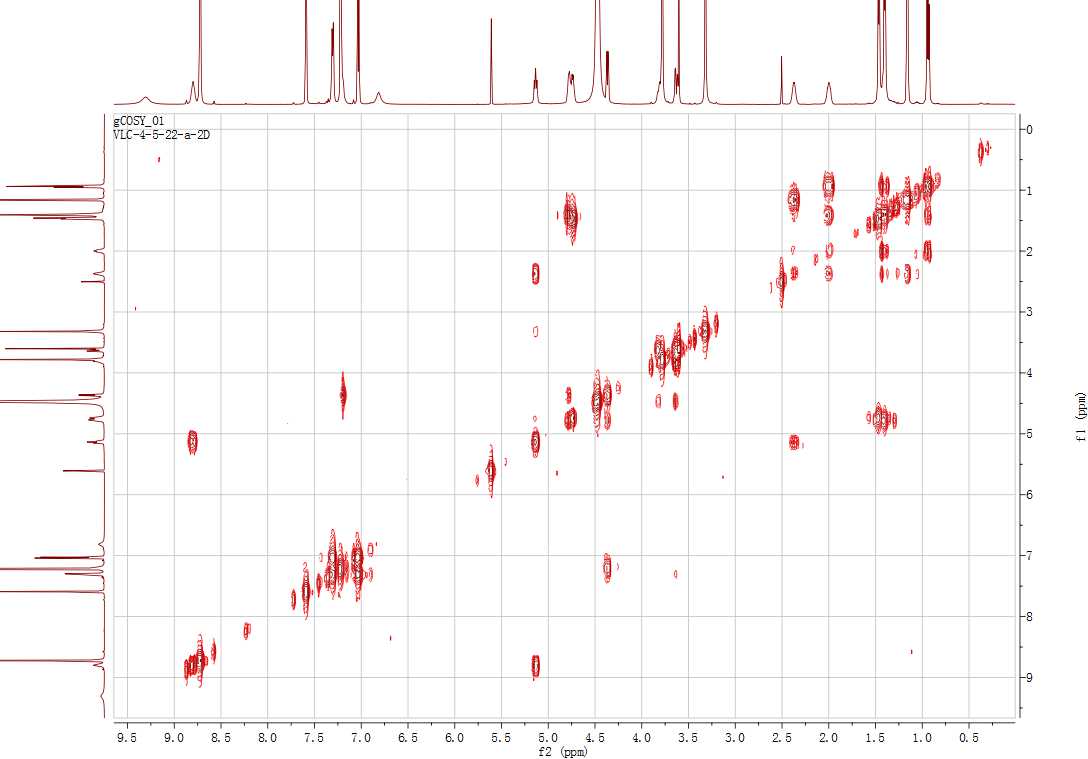


**Supplementary Figure 3.**

**
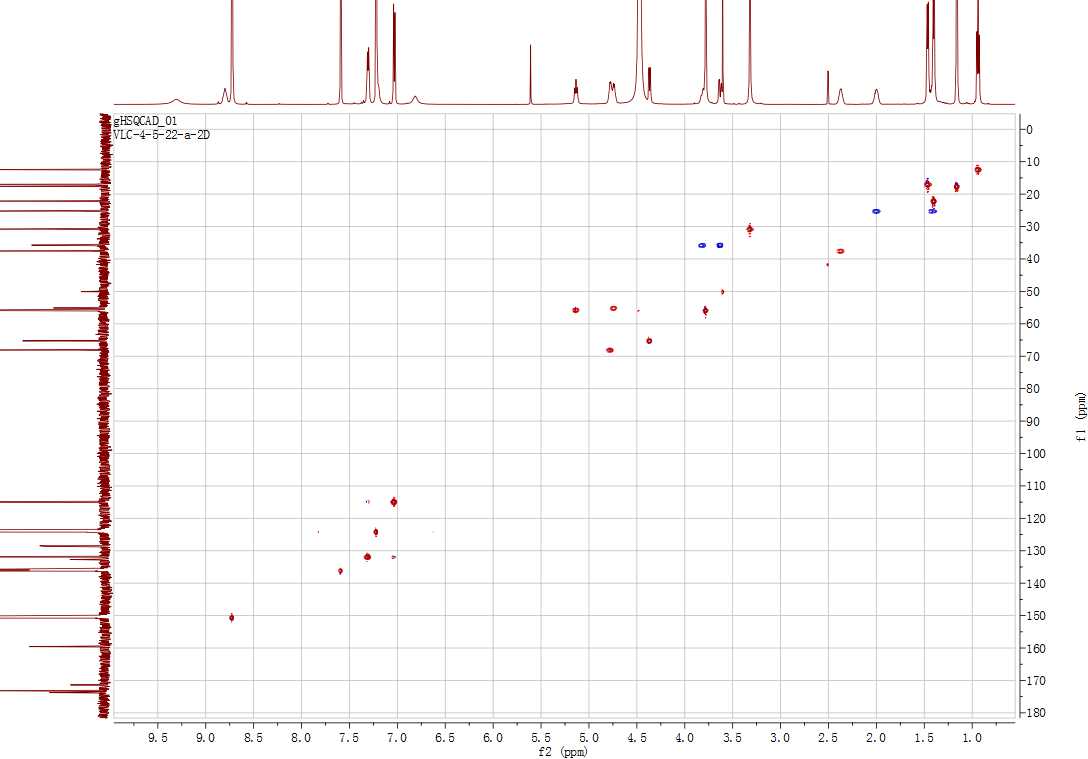
**

**Supplementary Figure 4.**

**
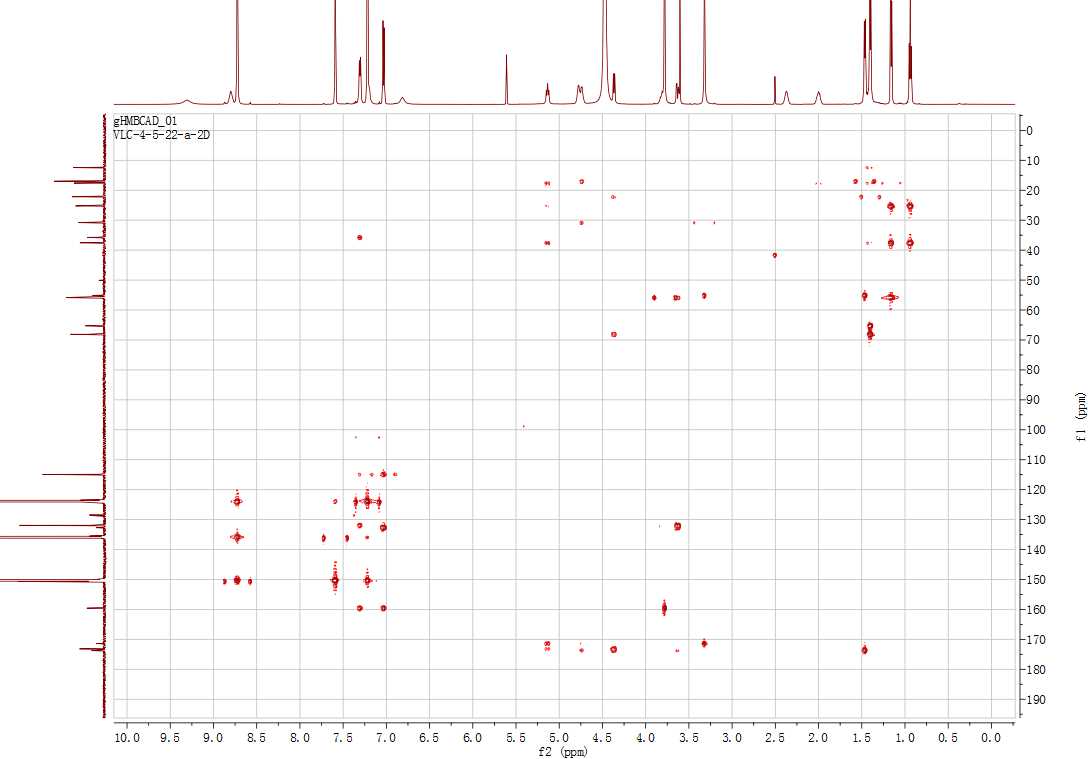
**

**Supplementary Figure 5.**

**
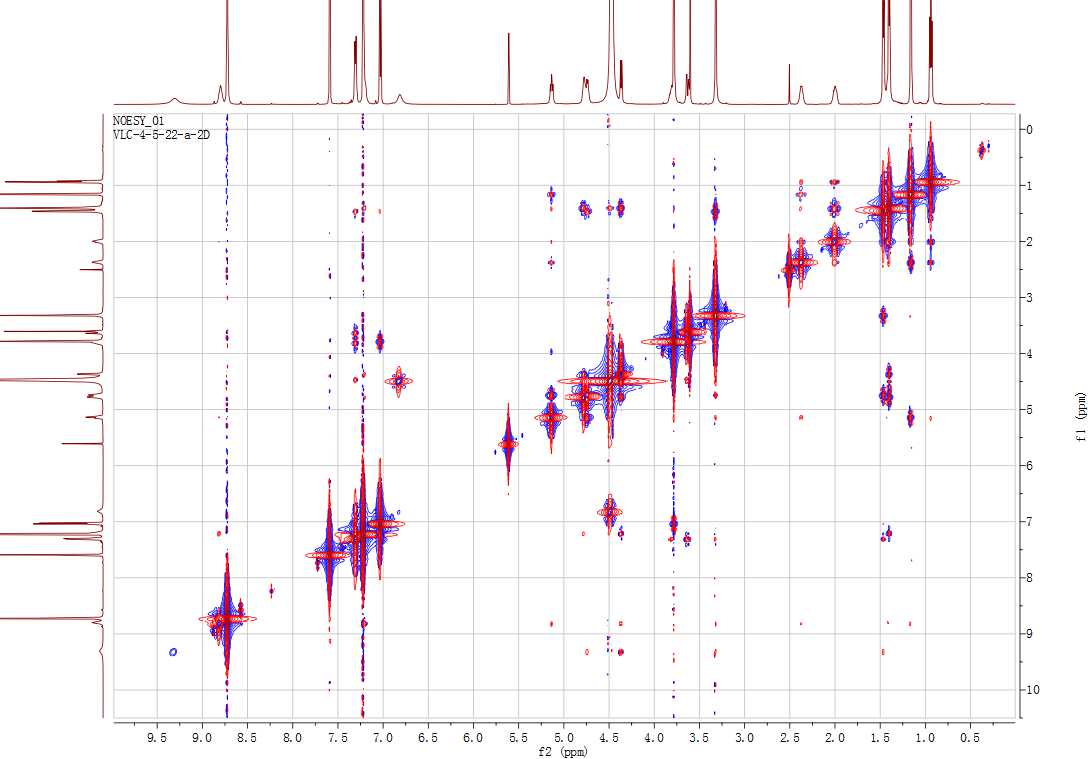
**

**Supplementary Figure 6.**

**
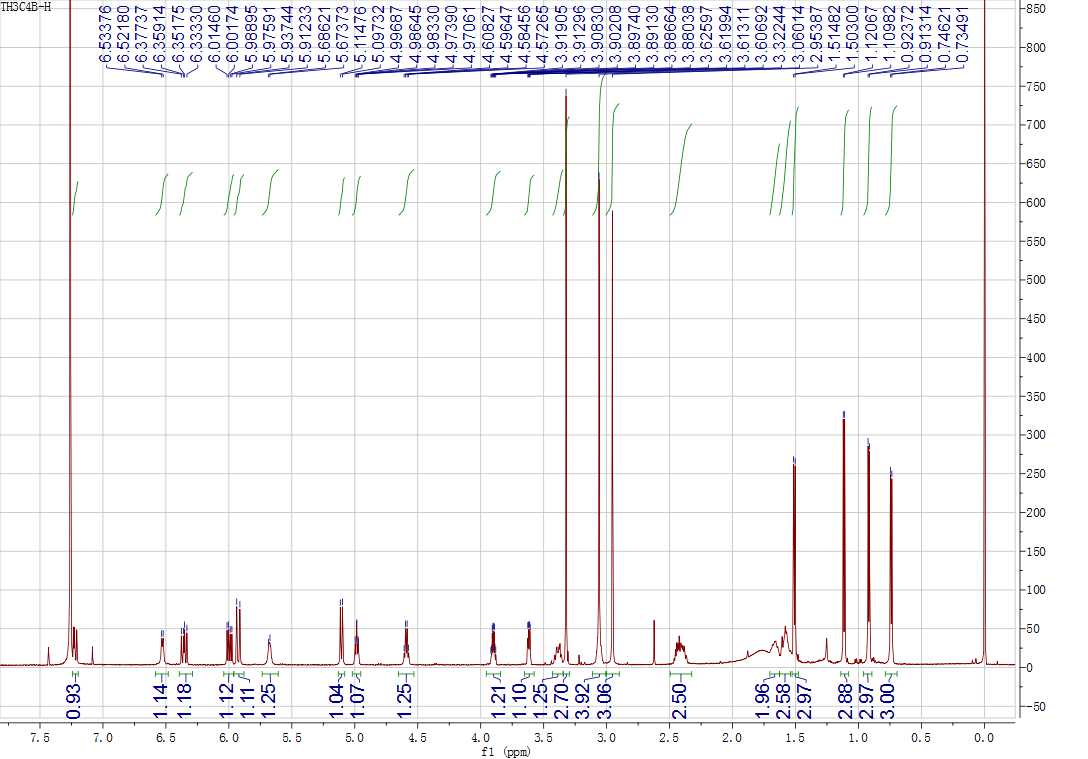
**

**Supplementary Figure 7.**

**
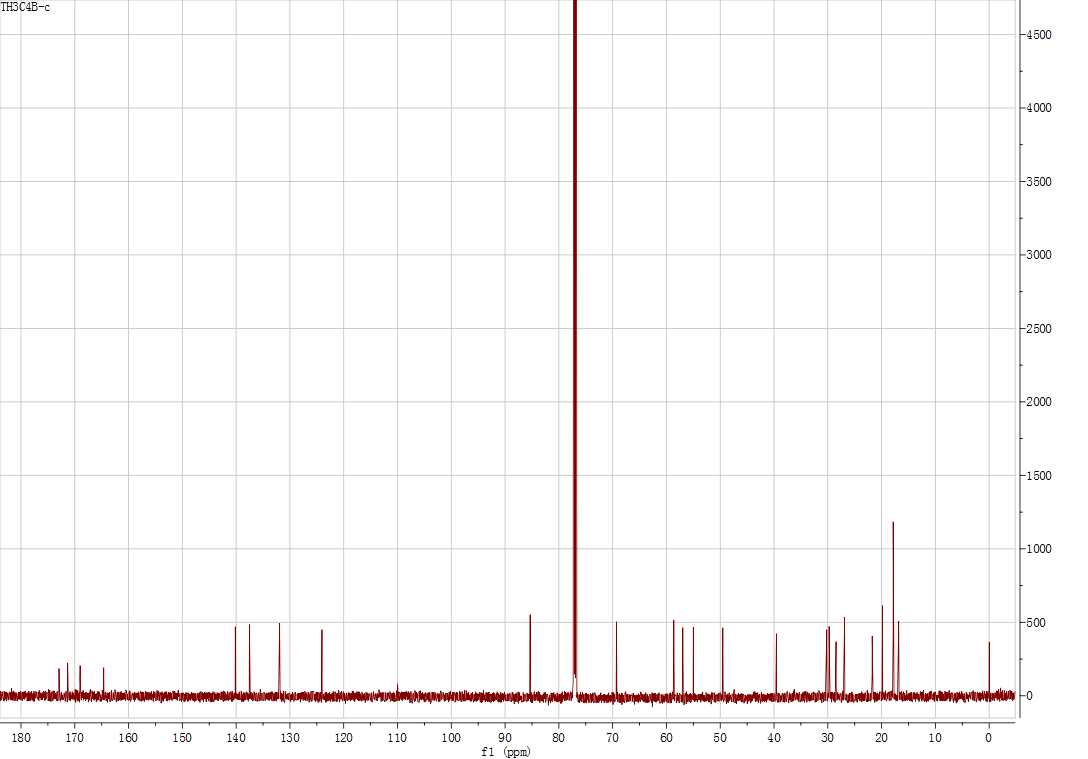
**

**Supplementary Figure 8.**


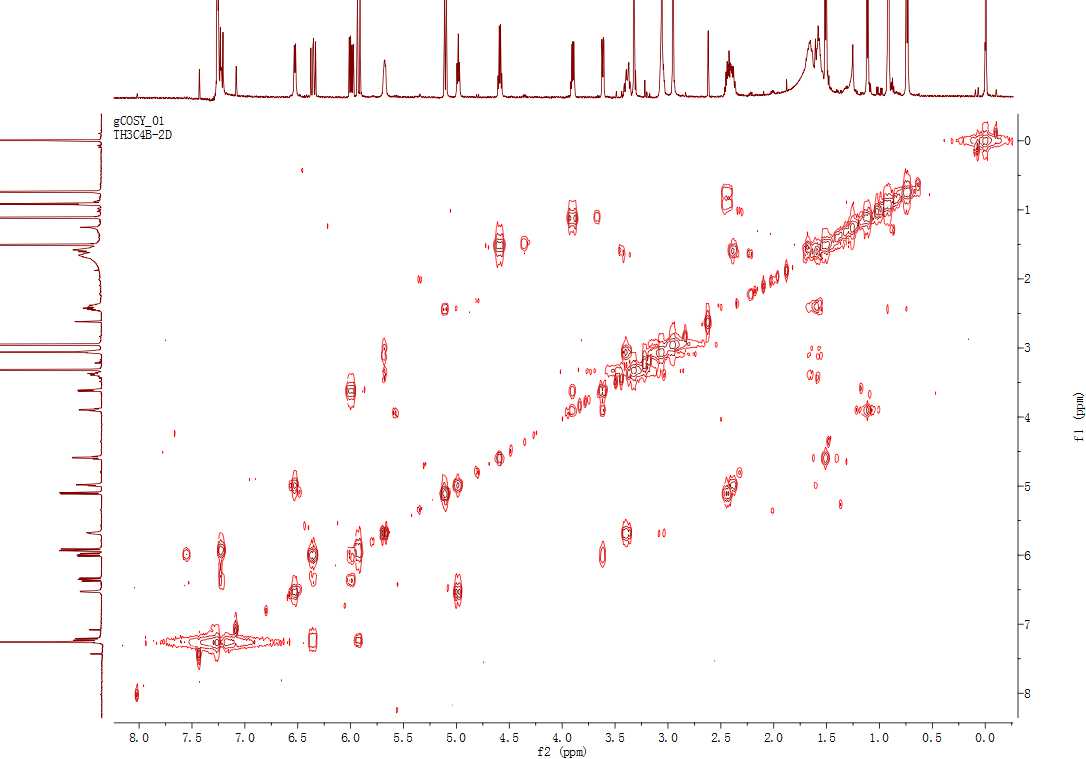


**Supplementary Figure 9.**


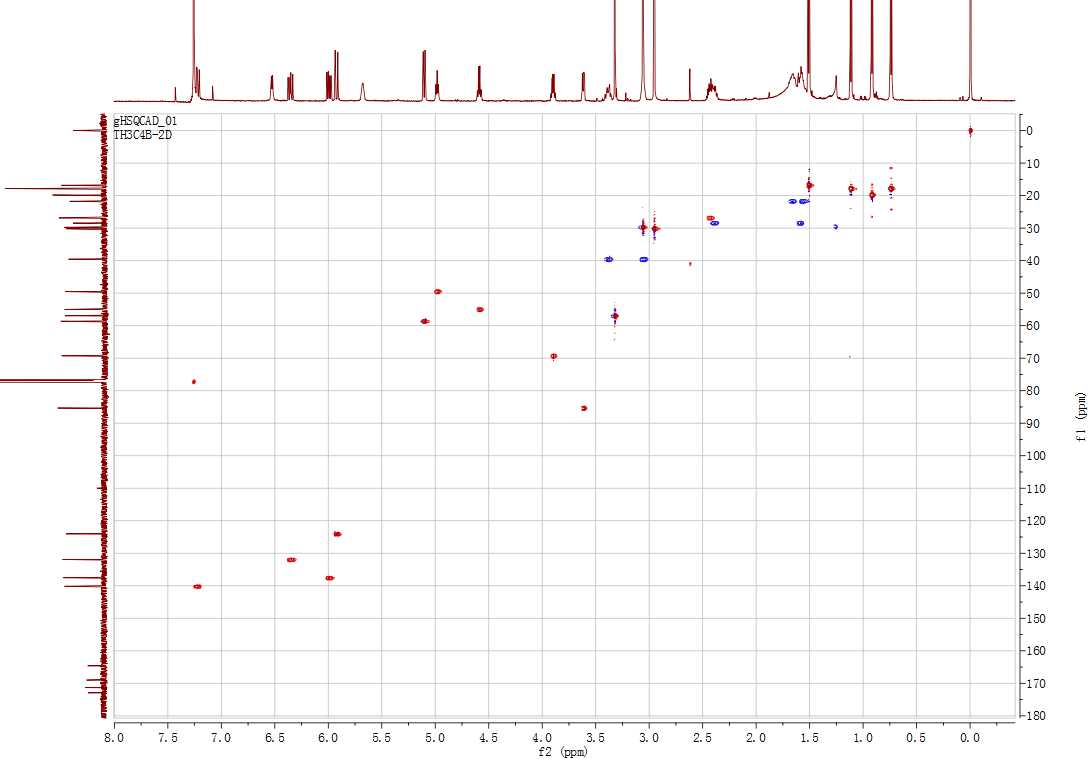


**Supplementary Figure 10.**


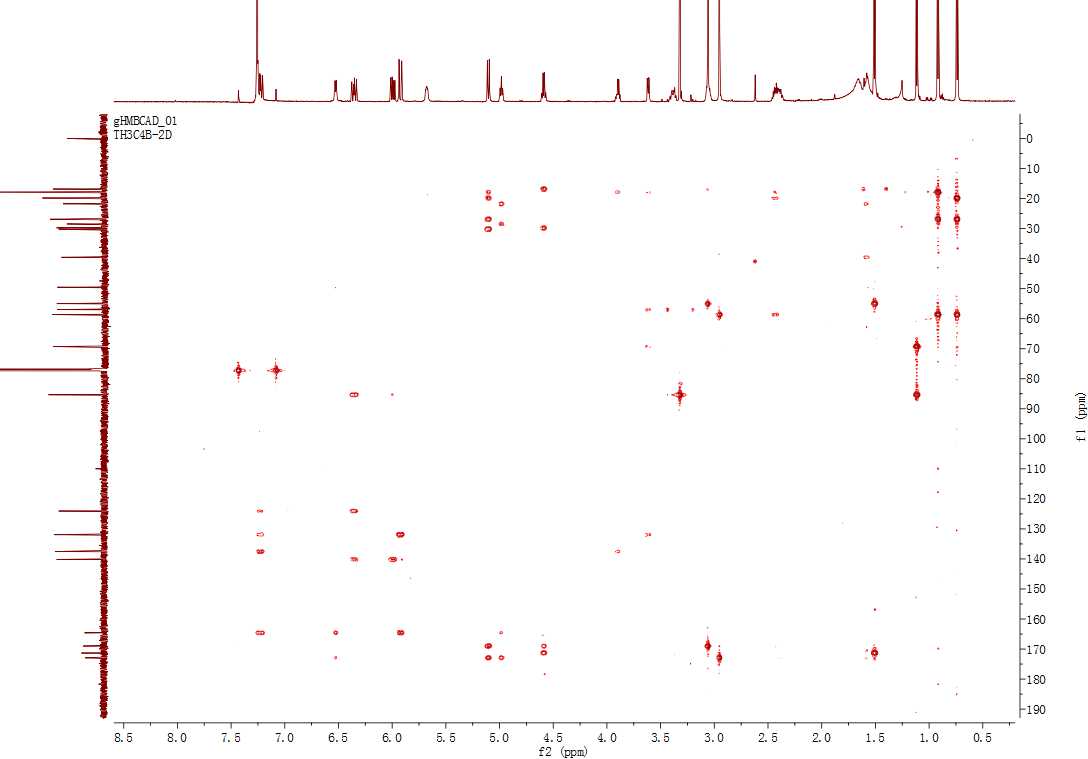


**Supplementary Figure 11.**


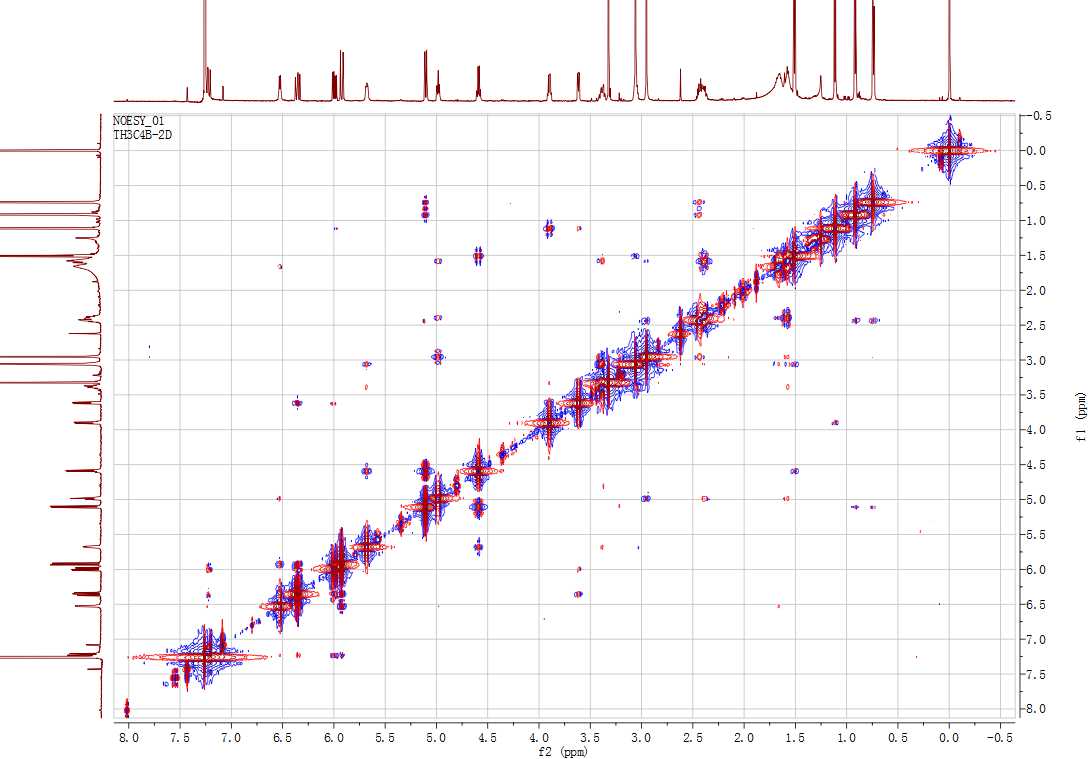


**Supplementary Figure 12.**


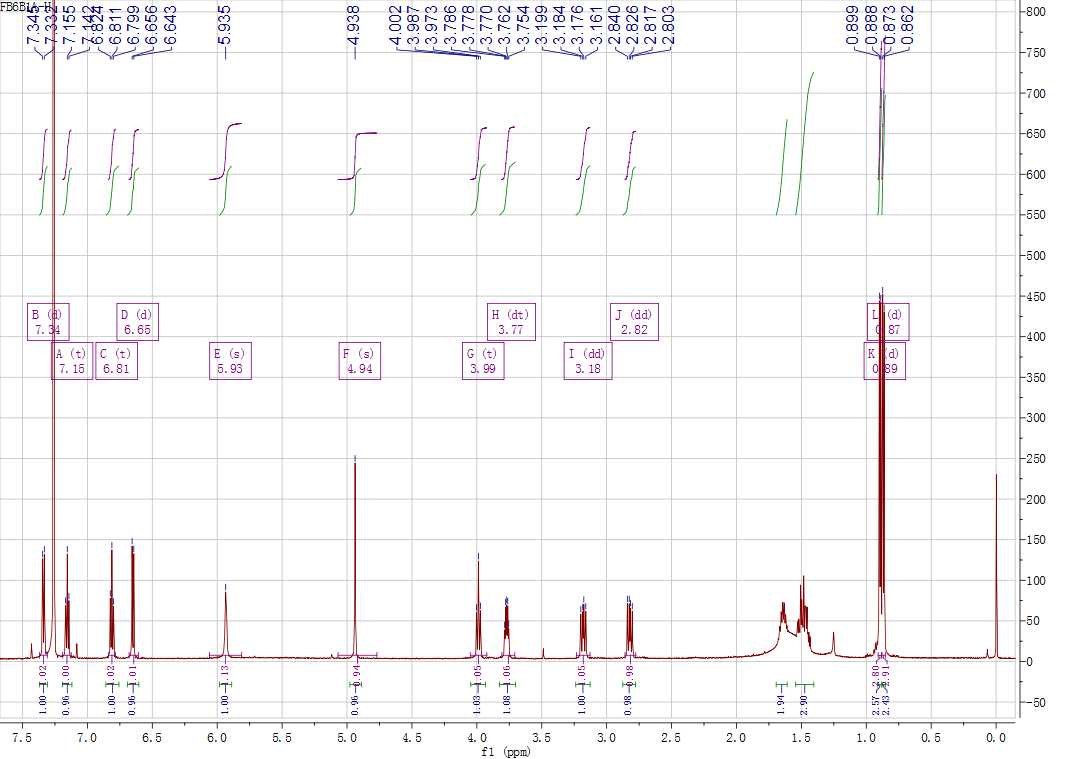


**Supplementary Figure 13.**


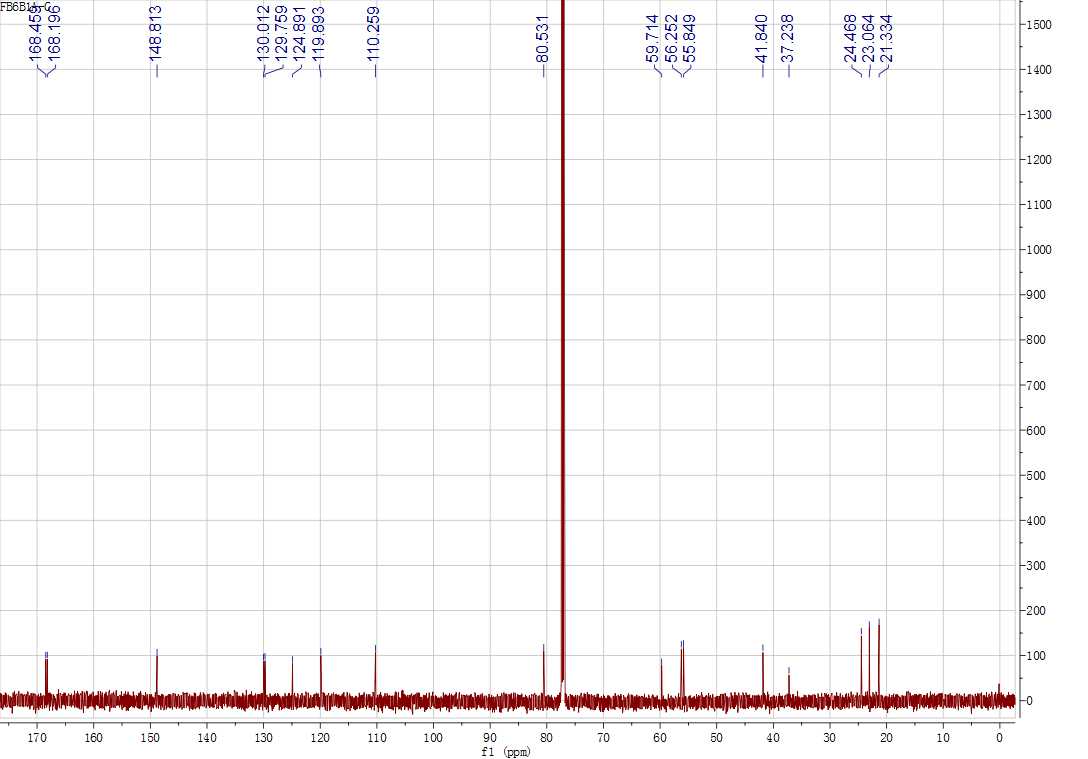


**Supplementary Figure 14.**


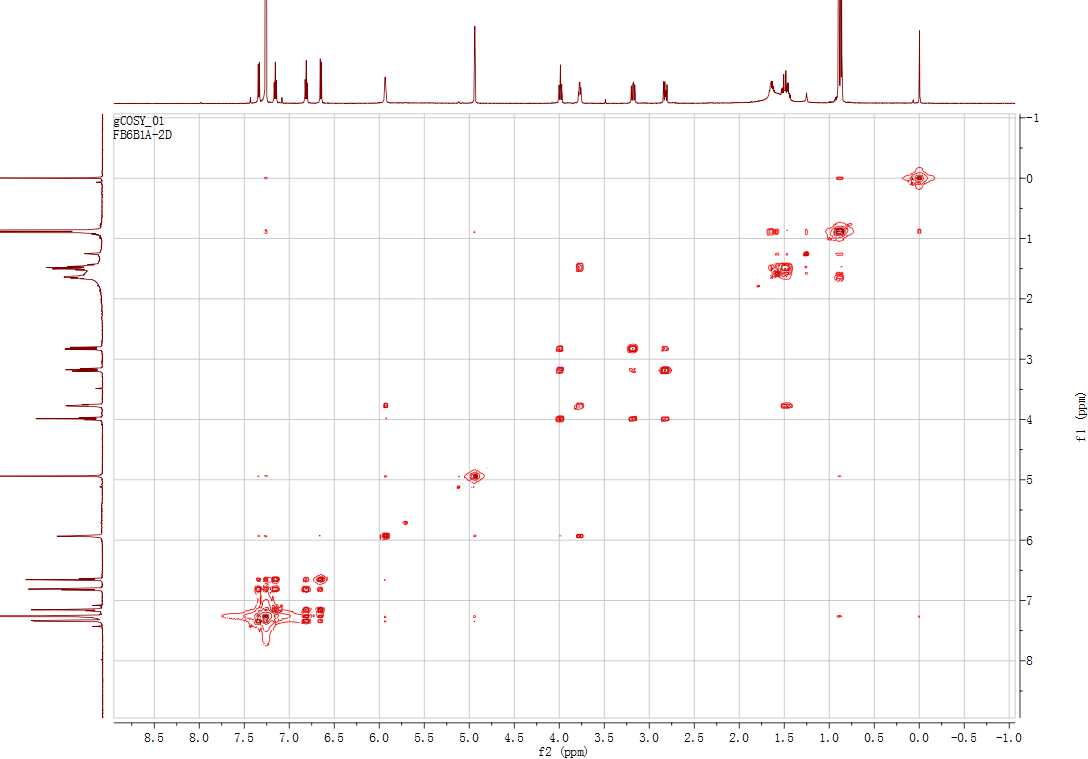


**Supplementary Figure 15.**


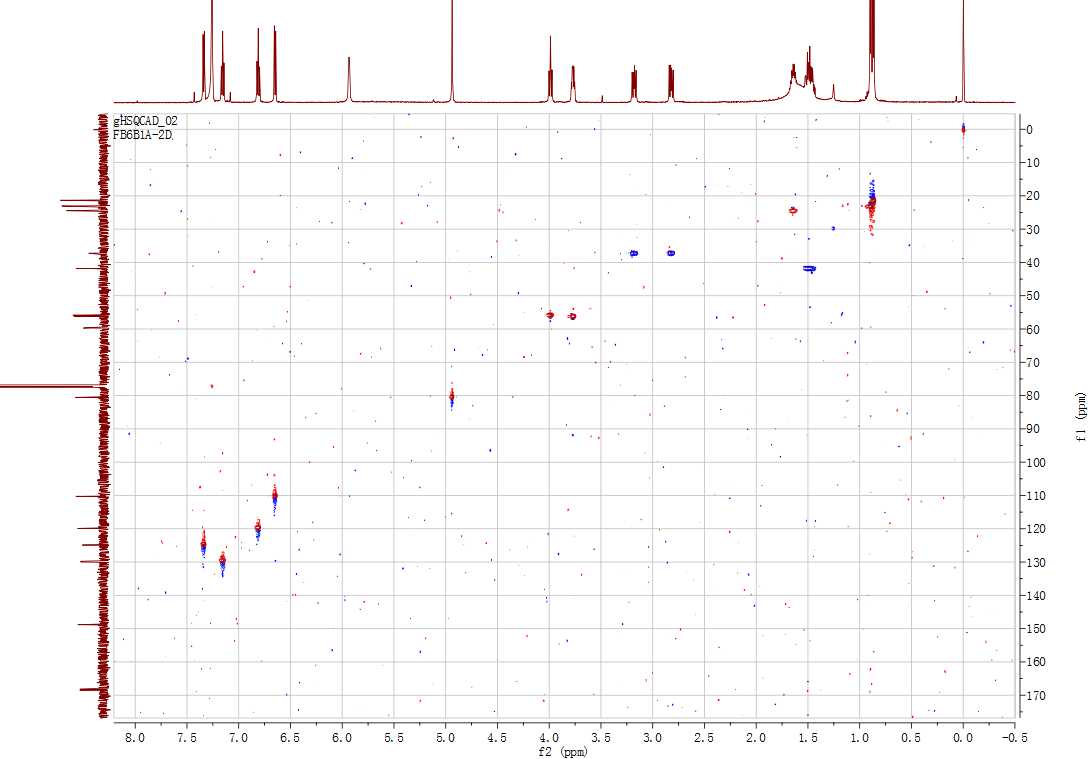


**Supplementary Figure 16.**


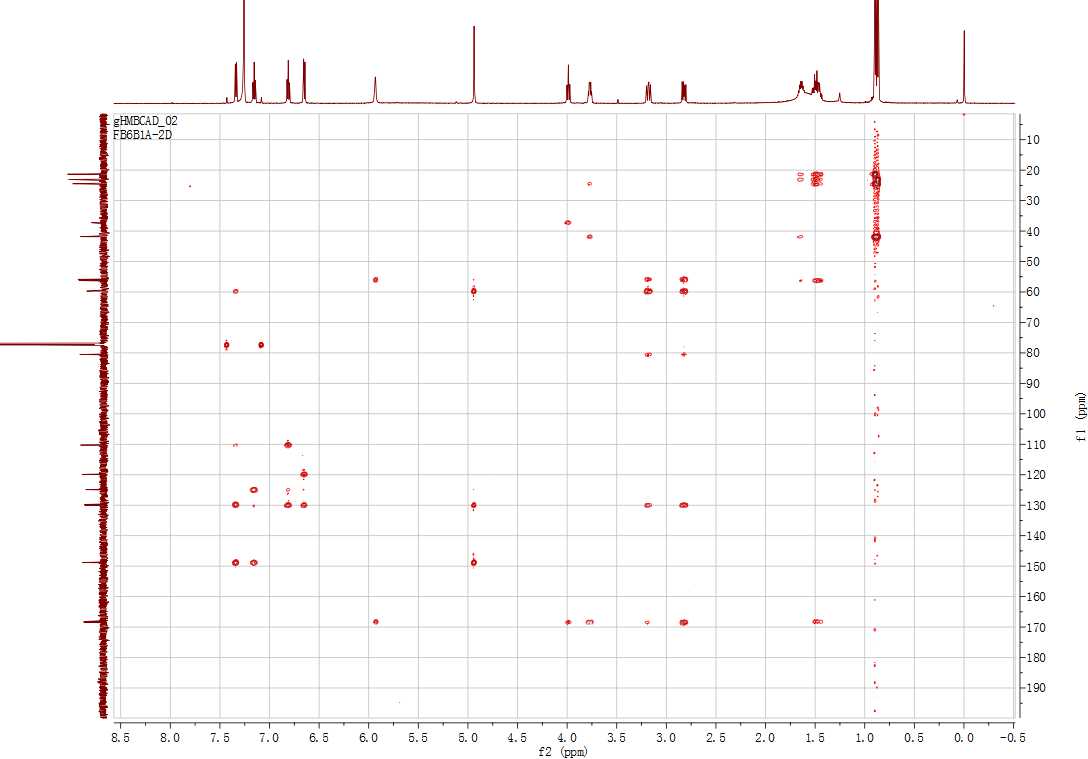


**Supplementary Figure 17.**


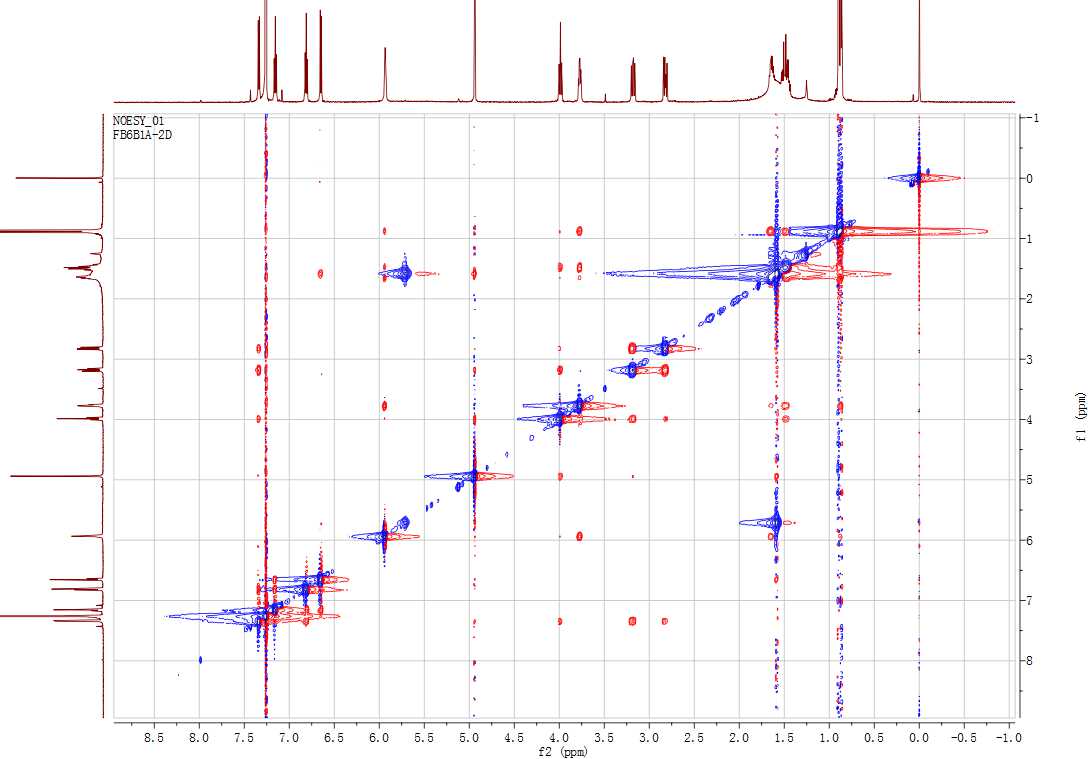


**Supplementary Figure 18.**


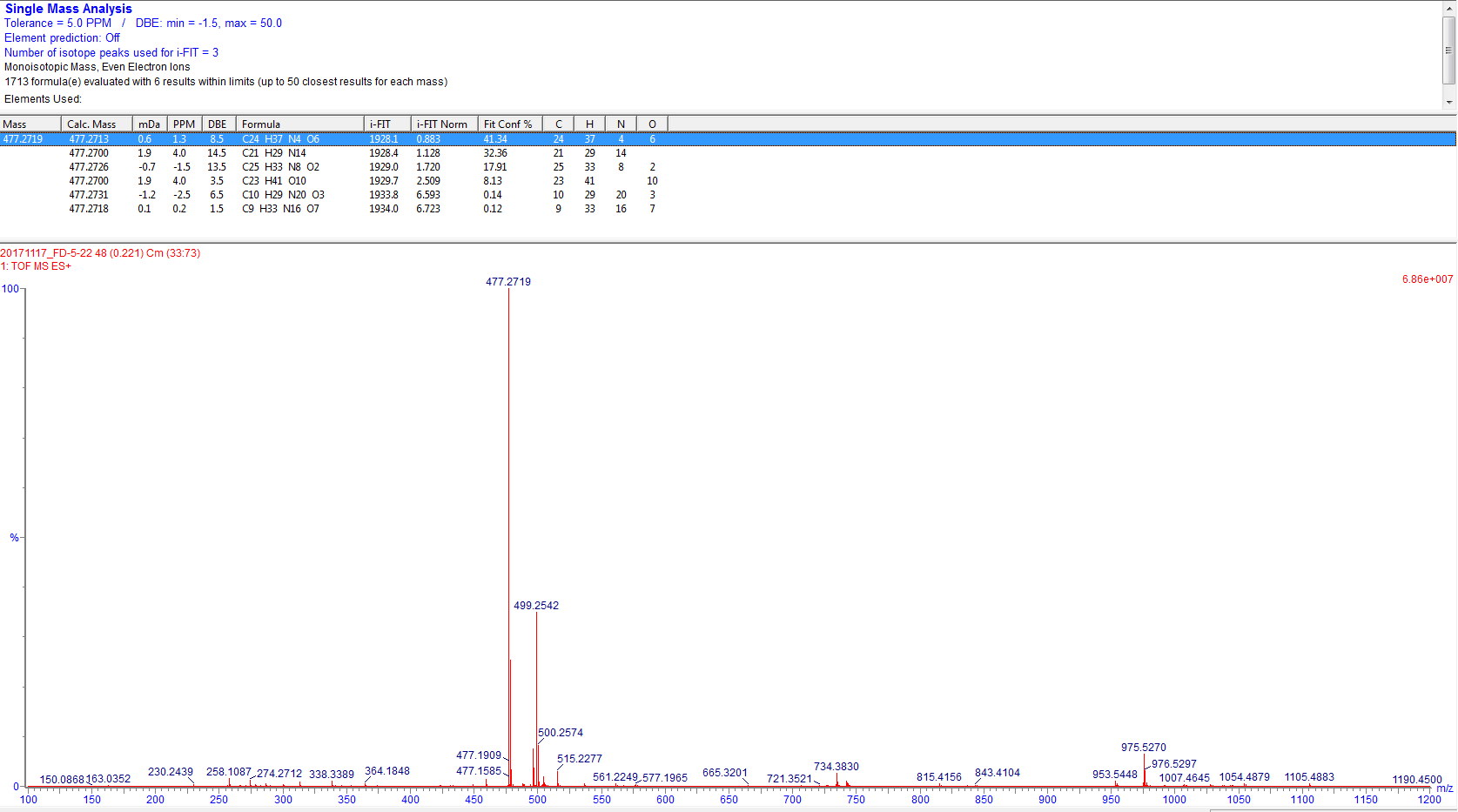


**Supplementary Figure 19.**


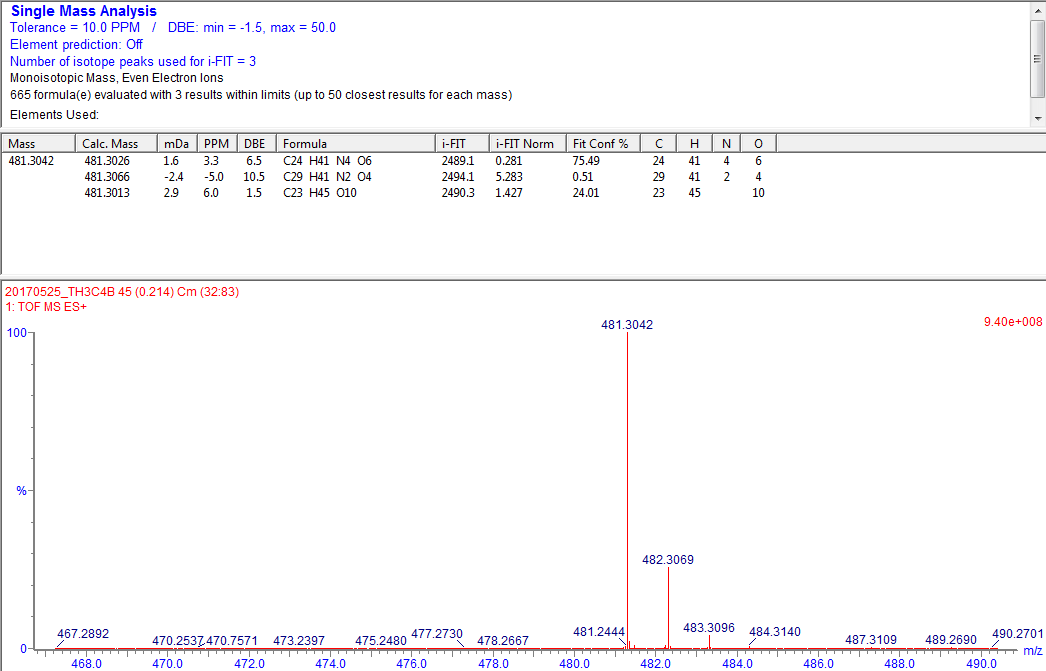


**Supplementary Figure 20.**


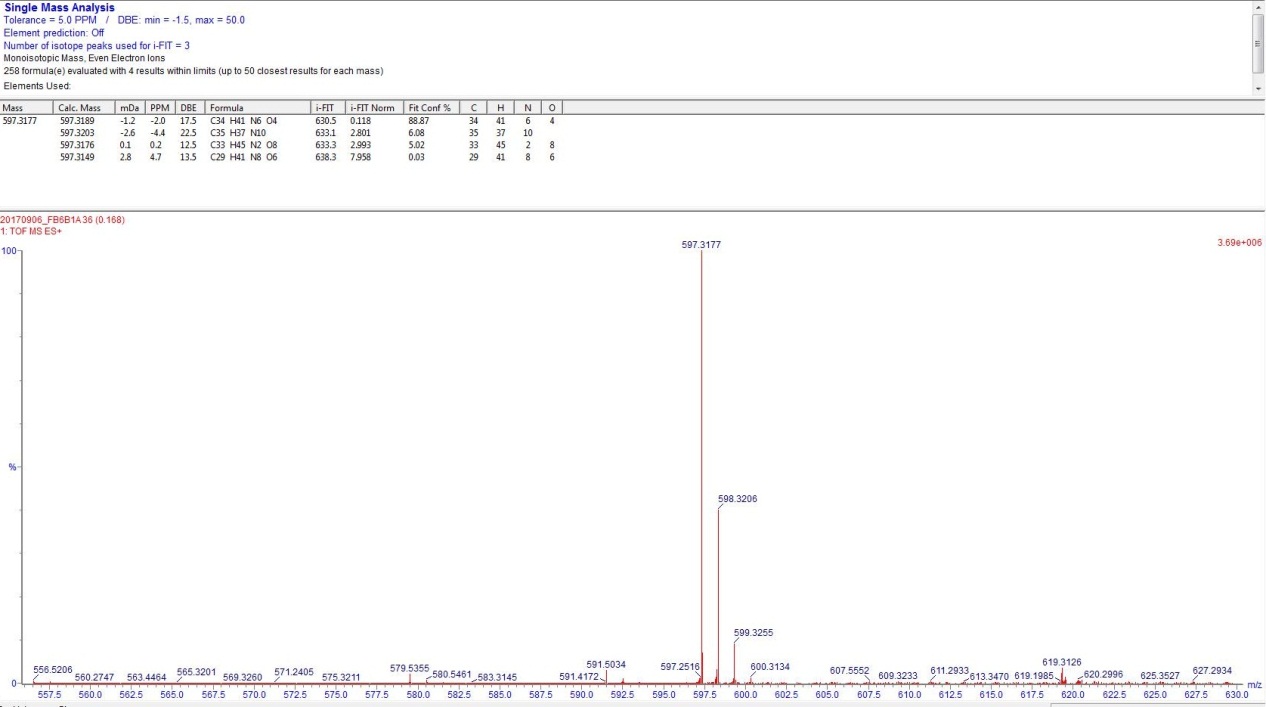


**Supplementary Figure 21.**


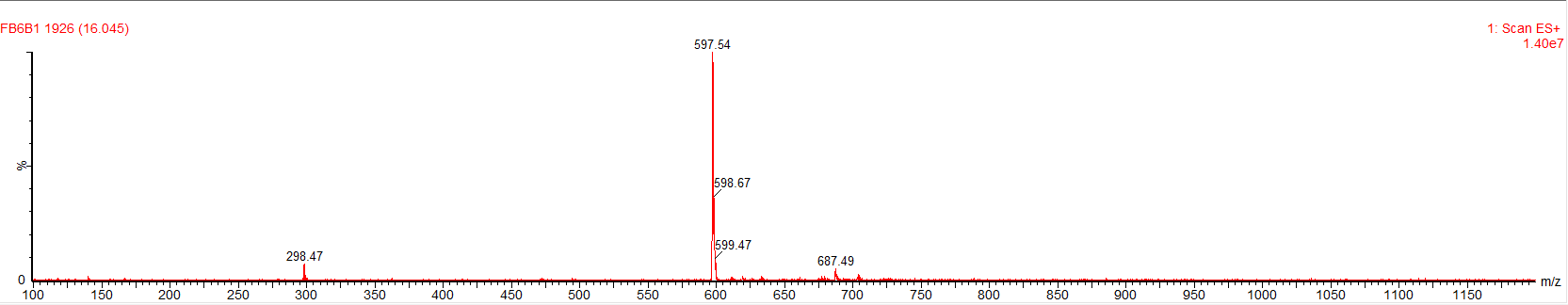


**Supplementary Figure 22.**

**
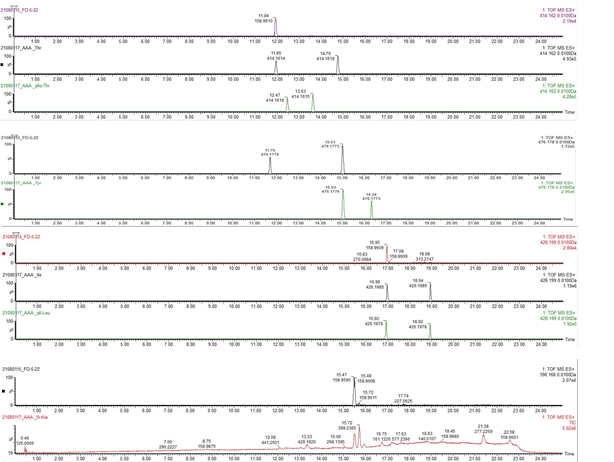
**

**Supplementary Figure 23.**


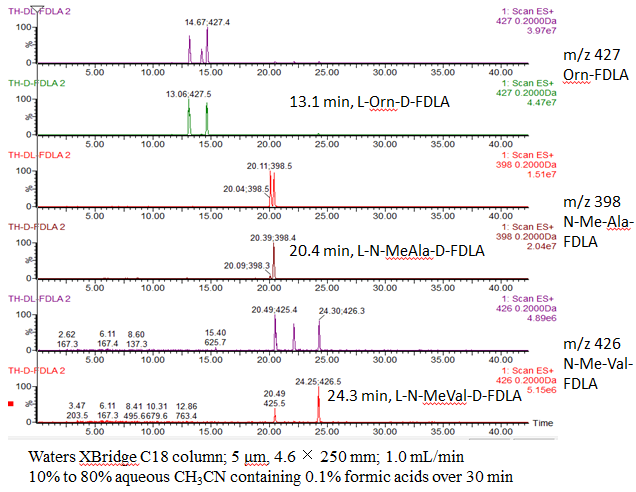


**Supplementary Figure 24.**


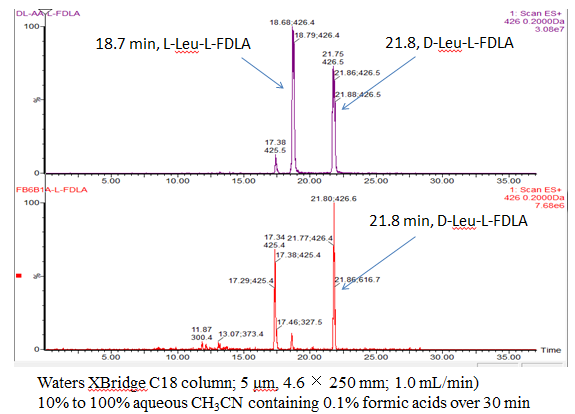


**Supplementary Figure 25.**
